# New Molybdenum(II) Complexes with α-Diimine Ligands: Synthesis, Structure, and Catalytic Activity in Olefin Epoxidation

**DOI:** 10.3390/molecules24030578

**Published:** 2019-02-06

**Authors:** Maria Vasconcellos-Dias, João Marreiros, Rita Sales, Vitor Félix, Paula Brandão, Carla D. Nunes, Maria José Calhorda

**Affiliations:** 1 Centro de Química e Bioquímica, DQB, Faculdade de Ciências, Universidade de Lisboa, Campo Grande, 1749-016 Lisboa, Portugal; mariavasconcellosdias@gmail.com (M.V.-D.); jtvrmarreiros@gmail.com (J.M.); ritansales@gmail.com (R.S.); cmnunes@fc.ul.pt (C.D.N.); 2 Department of Chemistry, CICECO—Aveiro Institute of Materials, University of Aveiro, 3810-193 Aveiro, Portugal; vitor.felix@ua.pt (V.F.); pbrandao@ua.pt (P.B.); 3 Centro de Química Estrutural, DQB, Faculdade de Ciências, Universidade de Lisboa, 1049-001 Lisboa, Portugal; 4 BioISI—Biosystems & Integrative Sciences Institute, DQB, Faculdade de Ciências, Universidade de Lisboa, Campo Grande, 1749-016 Lisboa, Portugal

**Keywords:** molybdenum, allyl complexes, isomers, crystal structures, iminomethylpyridine, epoxidation

## Abstract

Three new complexes [Mo(η^3^-C_3_H_5_)Br(CO)_2_{^i^PrN=C(R)C_5_H_4_N}], where R = H (IMP = *N*-isopropyl 2-iminomethylpyridine), Me, and Ph, were synthesized and characterized, and were fluxional in solution. The most interesting feature was the presence, in the crystal structure of the IMP derivative, of the two main isomers (allyl and carbonyls *exo*), namely the equatorial isomer with the Br trans to the allyl and the equatorial with the Br trans to one carbonyl, the position trans to the allyl being occupied by the imine nitrogen atom. For the R = Me complex, the less common axial isomer was observed in the crystal. These complexes were immobilized in MCM-41 (MCM), following functionalization of the diimine ligands with Si(OEt)_3_, in order to study the catalytic activity in olefin epoxidation of similar complexes as homogeneous and heterogeneous catalysts. FTIR, ^13^C- and ^29^Si-NMR, elemental analysis, and adsorption isotherms showed that the complexes were covalently bound to the MCM walls. The epoxidation activity was very good in both catalysts for the *cis*-cyclooctene and *cis*-hex-3-en-1-ol, but modest for the other substrates tested, and no relevant differences were found between the complexes and the Mo-containing materials as catalysts.

## 1. Introduction

**Scheme 1:**
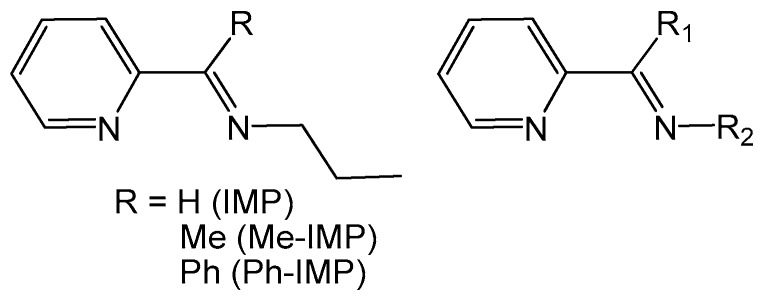
*N*-isopropyl 2-iminomethylpyridine (IMP) ligands.

*N*-isopropyl 2-iminomethylpyridine (^i^PrN=CHC_5_H_4_N, IMP) is an α-diimine ligand with lower symmetry than 2,2′-bipyridyl and, therefore, a promising candidate in the design of selective catalysts. Several authors have described the synthesis and characterization of complexes, such as [Mo(VI)O_2_Cl_2_(IMP)] [1], [Mo(II)(η^3^-C_3_H_5_)Cl(CO)_2_(R_1_,R_2_-IMP)], or [Mo(0)(CO)_4_(R_1_,R_2_-IMP)], with several R_1_ and R_2_ groups (Scheme 1) [2,3]. A few crystal structures have been determined by X-ray diffraction for the latter. The [Mo(CO)_4_(R_1_,R_2_-IMP)] complexes with R_1_ = H, Me, Ph and R_2_ = 2-X-Ph (X = COOH or SO_3_Na) were catalyzed the polymerization of methyl methacrylate (MMA) [4]. [Mo(CO)_4_(IMP)] was also immobilized in MCM-41 (MCM) with a spacer, and both the complex and the porous material were tested in the catalytic epoxidation of *cis*-cyclooctene [5]. More interestingly, [MoO_2_Cl_2_(IMP)] was a catalyst in the epoxidation of several substrates, including *R*-(+)-limonene, but selectivity issues were not addressed [1].

W(IV) complexes, [WBr_4_(R_1_,R_2_-IMP)] (R_1_ = H, R_2_ = Me, Et, ^n^Pr, tBu), were also synthesized and their magnetic properties studied by ^1^H-NMR [6]. Other metals were chosen to prepare complexes, namely Cu(I) with R_1_ = H and several R_2_ chains, which were active in MMA polymerization [7]. Palladium nanoparticles with IMP supported in functionalized silica were characterized and shown to be active in Suzuki-Miyaura cross coupling reactions and hydrogenation in water [8]. Another recent report addressed the selective oxidative cleavage of 1-octene catalyzed by arene Ru(II) complexes of R_1_,R_2_-IMP (R_1_ = H, R_2_ = functionalized chains) [9]. Besides catalysis, IMP ligands have also been studied owing to the photochemical properties exhibited by some of their derivatives, namely [Os_3_(CO)_19_(IMP)] clusters [10].

We synthesized Mo(II) complexes [Mo(X)_2_(CO)_3_(IMP)] (X = I, Br) and studied their catalytic activity in olefin epoxidation in the homogeneous phase and after their immobilization in MCM, having found an improved efficiency in the immobilized catalysts [11]. Since the complexes [Mo(η^3^-C_3_H_5_)X(CO)_2_(L_2_)] (X = halide, L_2_ = bidentate ligand) are often more active catalysts than [Mo(X)_2_(CO)_3_(L_2_)], we decided to prepare several derivatives of IMP with R_1_ = Me or Ph, as shown in Scheme 1 and designated as Me-IMP and Ph-IMP for facility. They were also immobilized in MCM, and the activity of the complexes and obtained materials was studied.

## 2. Results and Discussion

### 2.1. Chemical Studies: Complexes

**Scheme 2:**
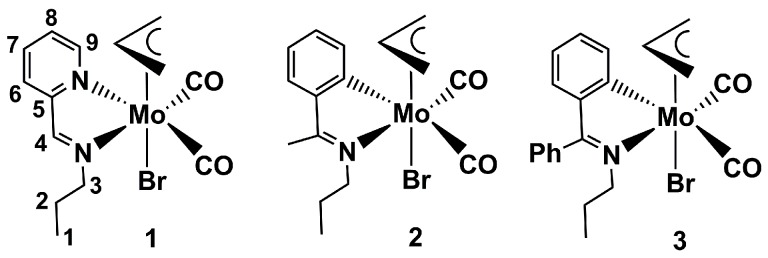
The three complexes (**1**, **2**, and **3)** with ligands IMP, Me-IMP, and Ph-IMP, respectively, with carbon, and respective hydrogens, numbering scheme (NMR).

Three new molybdenum complexes (**1**, **2**, and **3**) were obtained from reaction of [Mo(η^3^-C_3_H_5_)X(CO)_2_(MeCN)_2_] (X = Br, Cl) with ligands IMP, Me-IMP, and Ph-IMP, respectively, in dichloromethane after 6 h (Scheme 2). All the new complexes were characterized by elemental analysis, FTIR, ^1^H- and ^13^C-NMR spectroscopy, and complexes **1** and **2** also by single crystal X-ray diffraction (vide infra).

The FTIR spectra of complexes **1**–**3** showed the two characteristic νC≡O stretching modes of cis carbonyls at 1845 and 1942 cm^−1^(**1**), 1853 and 1936 cm^−1^(**2**), 1848 and 1924 cm^−1^(**3**), having slightly shifted from those of the parent compound [Mo(η^3^-C_3_H_5_)Br(CO)_2_(CH_3_CN)_2_] (1850 and 1947 cm^−1^). No peaks from νC≡N stretching mode, present in the latter complex at 2287 cm^−1^, were observed in complexes **1**–**3**. The frequency deviations of the νC=Nmodes of the bidentate ligands reflect the coordination to the metal. They vary from 1655 to 1647 cm^−1^(**1**), 1638 to 1593 cm^−1^(**2**), and 1629 and 1586 cm^−1^(**3**).

The ^1^H-NMR spectrum of **1** shows at least two isomers in solution. Two sets of peaks for H_anti_ protons at 1.25 and 1.44 ppm, two sets for the H_syn_ protons at 3.95 and 4.00 ppm, and one large peak at 4.46 assigned to H_meso_ were associated to the η^3^-C_3_H_5_ group. Three peaks at 1.09, 2.31, and 4.24 ppm were assigned to the hydrogens of the propyl chain of the coordinated IMP ligand, and those at 7.69 (H6), 7.86 (H7), 7.48 (H8), and 8.48 (H9) ppm, shifted from their position relative to the free ligand (7.87 (H6), 7.61 (H7), 7.98 (H8), and 8.70 (H9) ppm), belong to the pyridine ring. The signal of the H3 atom was observed at 8.24 ppm. Other signals were weaker and could not be completely assigned. Selected spectra for this and the other complexes are shown in Figures S1–S5 and Table S1 in Supporting Information (SI).

The signals for the carbon atoms in the ^13^C-NMR were also observed: 11.6 (C1), 23.5 (C2), and 72.7 (C3) for the propyl chain; 55.1–68.4 (C_allyl_) for the allyl; and 160.2 (C4), 152.7 (C5), 126.3 (C6), 137.7 (C7), 127.6 (C8), 150.2 (C9) for the iminopyridine.

**Scheme 3:**
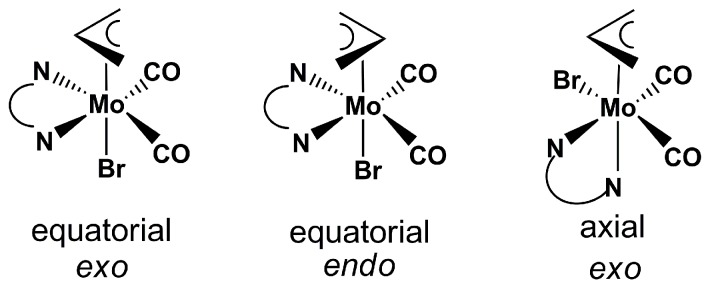
Main isomers of [Mo(η^3^-C_3_H_5_)Br(CO)_2_(L_2_)] complexes.

The isomers observed in the ^1^H-NMR spectrum arose from the fluxional behavior of the allyl ligand, as sketched in Scheme 3. The two isomers present in higher amounts should be the equatorial and the axial ones (exo) and their interconversion proceeded by allyl rotation [12,13]. Their ratio depended on the steric and electronic effects of the substituents.

The ^1^H-NMR spectrum of **2** is very similar—with two sets of three signals corresponding to the allyl protons at 4.41 and 4.80 ppm (two multiplets), 4.26 and 4.11 ppm (two doublets), and 1.61 and 1.49 ppm (two doublets), which were assigned to H_meso_, H_syn_, and H_anti_ protons—, indicating the presence of two isomers in solution. The signal of H4 was replaced by the signal of the three methyl hydrogens.

The ^1^H and ^13^C{1H} NMR were studied in more detail using COSY and HMQC experiments (SI, Figures S3–S5). The COSY spectrum allowed the identification of three sets of signals, from the four doublets (1.16, 1.25, 1.34, and 1.48 ppm H_anti_), three signals (2.21, 3.14, and 3.28 ppm H_syn_), and three multiplets (3.58, 3.76, and 3.95 ppm H_meso_). From the correlation, it was possible to assign one set for the equatorial isomer (exo), another for the equatorial isomer (endo), and the third—comprising two doublets, for the H_anti_—for the axial isomer (Scheme 2). Indeed, the axial isomer was less symmetric and the two H_anti_ were more inequivalent [14].

**Figure 1:**
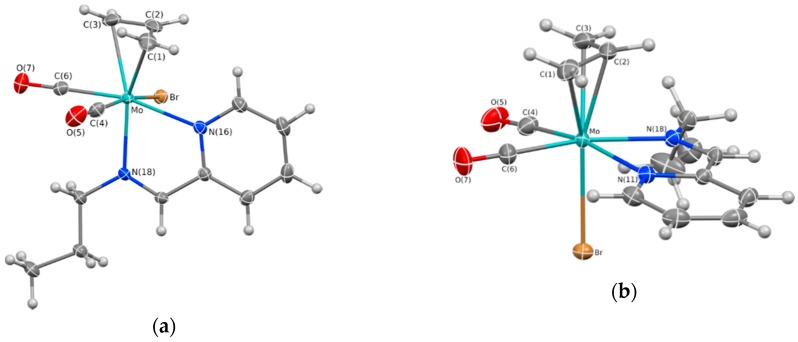
Molecular structures of the isomers found in the crystal structure of [Mo(η^3^-C_3_H_5_)Br(CO)_2_(IMP)] (**1**) with the thermal ellipsoids drawn at the 50% probability level: (**a**) axial-exo; (**b**) equatorial-exo.

**Figure 2:**
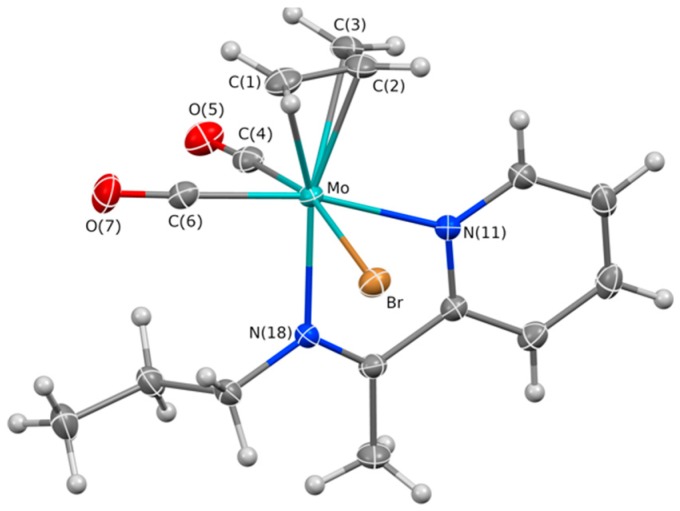
View of the asymmetric unit of [Mo(η^3^-C_3_H_5_)Br(CO)_2_(Me-IMP)] (**2**) showing the exo-axial binding arrangement adopted and thermal ellipsoids drawn at the 50% probability level.

The crystal structures of [MoBr(η^3^-C_3_H_5_)(CO)_2_(IMP)] (**1**) and [MoBr(η^3^-C_3_H_5_)(CO)_2_(Me-IMP)] (**2**) were determined by single crystal X-ray diffraction, and are shown in Figure 1 and Figure 2, respectively. Selected bond distances and angles for **1** and **2** are given together in Table 1.

The selected bond distances and angles for **1** and **2** are given together in Table 1. In both complexes, the allyl ligand adopted an exo conformation, with the terminal allylic carbons over the carbonyls, as sketched in Scheme 3. The coordination geometry around the molybdenum center can be described as pseudo octahedral, with the centroid of the allyl ligand and the two carbons from the cis-carbonyl groups determining a fac arrangement, as previously found in many structures [14]. Moreover, the asymmetric unit of **1** is composed of two geometric isomers with the ligands exhibiting different spatial dispositions in the metal coordination sphere. Thus, in the axial isomer, a nitrogen donor of IMP ligand occupied an axial coordination site (Figure 1, left side), while in the equatorial isomer, the two nitrogen donors of this chelating ligand defined the equatorial coordination plane together with the two carbonyl ligands (Figure 1, right side). The existence of these two isomers in the solid state were in agreement with the ^1^H-NMR structural findings observed in the solution.

The Mo-N(11) and Mo-N(18) distances in the equatorial isomer were 2.2995(16) and 2.2072(15) Å, respectively. These three atoms, with an N(11)-Mo-N(18) angle of 73.36(4)˚, and two other carbon atoms formed a five-membered ring. A bromine atom occupied the remaining position in the metal coordination sphere, trans to the allyl ligand, with a Mo-Br distance 2.6961(2) Å. The Mo-N(11) and Mo-N(18) distances increased to 2.2371(12) and 2.3266(16) Å, respectively, in the axial isomer, leading to an N(11)-Mo-N(18) angle of 72.74(4)˚. The Mo-N(18) distance became significantly longer when the N(18) atom was trans to the allyl instead of trans to one CO. The bromine atom, which occupied now a position trans to one CO, made a shorter Mo-Br bond of 2.6408(2) Å. The spatial disposition of the ligands in complex **2** (Figure 2) was equivalent to that observed in the axial exo isomer of the analogous **1**, as indicated by the comparable bond lengths and angles presented in the Table 1 for these two complexes.

Moreover, the values of Mo-N distances and the N-Mo-N chelating angle determined for **1** and **2** agreed with those found for analogous Mo(η^3^-C_3_H_5_)XCO)_2_ complexes (axial isomers) with bis(4-Y-phenyl)-acenaphthenequinone diimine ligands: 2.277(4) and 2.229(4) Å, respectively, and an N(11)-Mo-N(18) bite angle of 72.7(1) °, for the derivative with X = Cl and Y = Cl [15]; and 2.2863(17), 2.2948(16) Å and 2.2298(16), 2.2314(16) Å and a bite angle of 73.41(6), 73.33(6) ° in the two enantiomers of the unit cell of the derivative with X = SCN and Y = H [16].

### 2.2. Chemical Studies: Materials

**Scheme 4:**
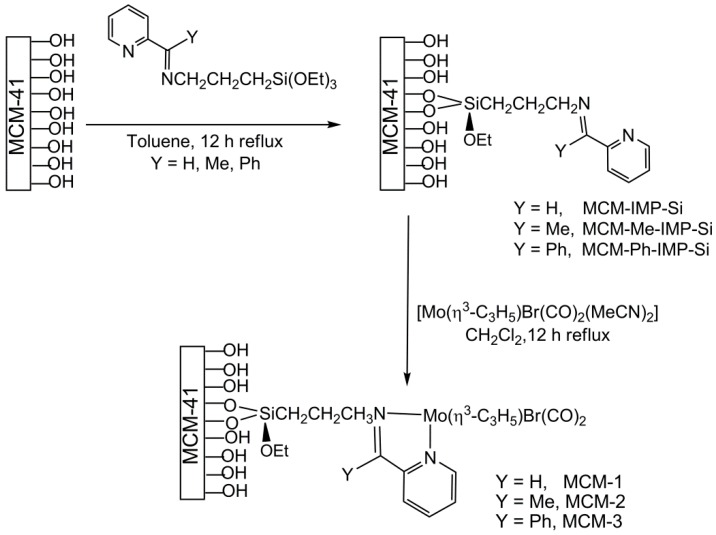
Procedure for the immobilization of complexes **1**–**3** in MCM.

The three new complexes, **1**, **2** and **3,** were immobilized in MCM-41 (MCM) according to the reported procedure [16] described in Scheme 4. The MCM-41 was synthesized by a template approach [17].

In the first step, the silylated ligands (IMP-Si, Me-IMP-Si, and Ph-IMP-Si) reacted with the MCM surface OH groups to afford the ligand functionalized materials MCM-IMP-Si, MCM-Me-IMP-Si, and MCM-Ph-IMP-Si. The second step consisted of the reaction between these materials and the precursor complex [Mo(η^3^-C_3_H_5_)Br(CO)_2_(MeCN)_2_] with formation of three Mo-containing materials (MCM-1, MCM-2, and MCM-3).

CHN analyses of MCM-1, MCM-2, and MCM-3 materials showed 6.01% C, 1.92% H, and 1.64% N for MCM-1; 5.08% C, 1.90% H, and 2.03% N for MCM-2; and 14.16% C, 2.26% H, and 2.68% N for MCM-3. Based on the N content, these results indicated that the loading of the Y-IMP ligand inside the pores of the different materials was found to be 0.59, 0.72, and 0.96 mmol·g^−1^, for the MCM-1, MCM-2, and MCM-3 materials, respectively. In addition, the Mo contents were determined to be 4.90, 4.79, and 3.45% for MCM-1, MCM-2, and MCM-3, corresponding to loadings of 0.51, 0.50, and 0.36 mmol_Mo_·g^−1^, respectively. The latter results showed that all Mo cores were coordinated to the surface-grafted ligands, and not directly to the MCM wall, as the loading of ligands was higher in all materials, as shown in Scheme 4.

All materials were characterized by powder XRD, Diffuse Reflectance Infrared (DRIFT), and ^29^Si and ^13^C CP MAS NMR. Sorption/desorption N_2_ isotherms were also determined for estimation of textural parameters. All characterization features are consistent with those reported in related mesoporous materials [17].

**Figure 3:**
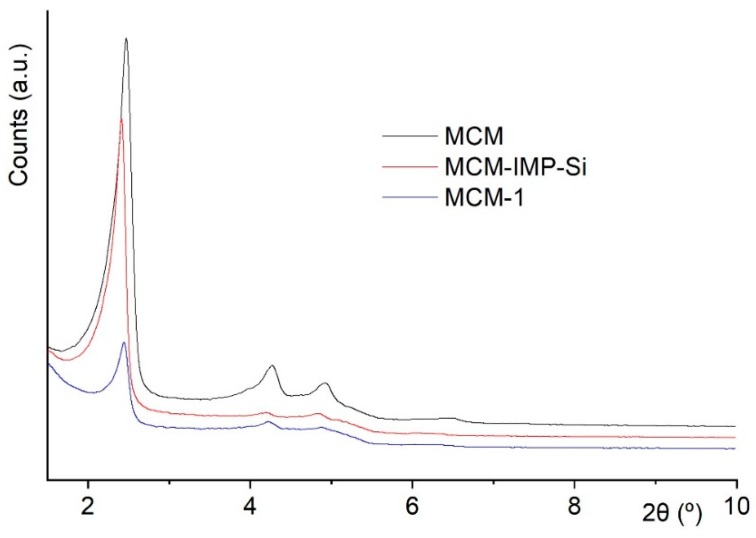
XRD powder patterns of materials MCM, MCM-IMP-Si, and MCM-1.

The powder X-ray patterns of the materials containing the ligands IMP-Si, Me-IMP-Si, and Ph-IMP-Si affording materials MCM-IMP-Si, MCM-Me-IMP-Si, and MCM-Ph-IMP-Si, respectively, are presented in Figure 3 for the IMP group. The powder pattern of the calcined parent material MCM-41 clearly shows four reflections in the 2*θ* range 2–10°, indexed to the (100), (110), (200), and (210) reflections of a hexagonal unit cell [18]. The constant of the lattice was obtained from the *d* value of the (100) reflection (35.6 Å) and found to be *a* = 41.1 (2*d*_100_/√3). The functionalization of the MCM-41 material walls with the IMP-Si ligand did not significantly change the 2*θ* angles characteristic of the more important reflections, confirming that the material retained the long-range hexagonal symmetry present in the parent MCM-41. The same trend was observed for materials MCM-Me-IMP-Si and MCM-Ph-IMP-Si. After functionalization of the MCM-Me-IMP-Si and MCM-Ph-IMP-Si materials with the Mo(II) core, the materials MCM-1, MCM-2, and MCM-3 still showed three reflections.

There was a slight deviation of the maxima toward lower 2*θ* values, as compared to the parent MCM. In material MCM-IMP-Si, the *d*_100_ value and the related a parameter increased with incorporation of the ligand (*d*_100_ = 36.5 Å and *a* = 42.1 Å for MCM-IMP-Si) and the metal fragment (*d*_100_ = 36.2 Å and *a* = 41.8 Å for MCM-1). Similar results were obtained for the materials MCM-Me-IMP-Si, MCM-Ph-IMP-Si, MCM-2, and MCM-3. Other data characterizing the materials are included in Table 2, summarizing the evolution of their relevant textural properties until the immobilization of Mo(II) precursor complex. The observed peak intensity reduction is common to all materials, being even more significant in the materials with the Mo cores. This is not due to a crystallinity loss, but rather to an X-ray scattering contrast reduction between the silica walls and the pore filling material. This has been observed for other types of materials and has been well described in the literature [19,20].

**Figure 4:**
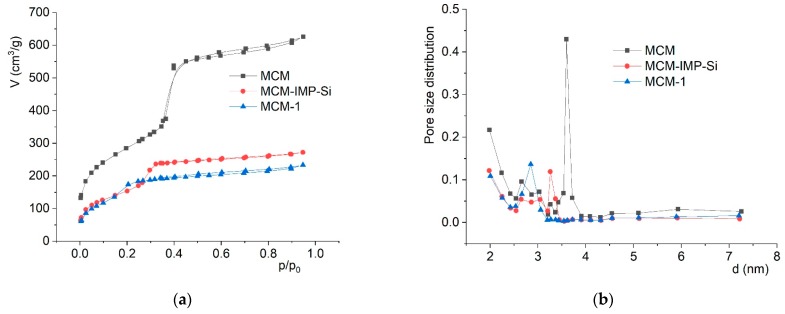
(**a**) Nitrogen adsorption isotherms at 77 K and (**b**) pore size distribution (PSD) curves of MCM, MCM-IMP, and MCM-1.

Nitrogen sorption/desorption studies at 77 K were performed and revealed that all materials exhibited a reversible type IV isotherm (Figure 4), typical of mesoporous solids (pore width between 2 nm and 50 nm, according to IUPAC) [21]. The calculated textural parameters (*S_BET_* and *V_P_*) of the synthesized materials (Table 2) agreed with the literature data [22,23]. The nitrogen adsorption curves displayed a steep and sharp capillary condensation/evaporation step in the parent MCM-41 at 0.35–0.4 relative pressure range, indicating a uniform pore size distribution (PSD). After functionalization with the ligand, the isotherm of the new MCM-IMP-Si showed a much smaller step. The lower N_2_ uptake reflected a decrease (Table 2) in both the volume *V_P_* (57%) and surface area *S_BET_* (46%). In the case of MCM-Me-IMP-Si, the obtained decrease was similar to that achieved for the previous material, with changes in *S_BET_* and *V_P_* of 35% and 57%, respectively. For MCM-Ph-IMP-Si, similar results were also obtained. These findings indicated that the ligand immobilization on the internal silica surface was accomplished (Figure 4, Table 2). For the materials with the Mo(II) core, the *S_BET_* and *V_P_* decrease relative to MCM was 47% and 63%, 50% and 74%, and 47% and 65% for MCM-1, MCM-2, and MCM-3, respectively.

These results agreed with the *p*/*p*_0_ coordinate decrease in the isotherm inflection points after post-synthesis treatments [24]. Furthermore, the maximum of the PSD curve (Figure 4) determined by the BJH method, *d_BJH_*, for MCM materials, decreased from 36.0 Å to 28.5, 26.7 Å, and from 32.8 Å to 26.6 Å (Table 2) for MCM-1, MCM-2, and MCM-3, respectively. These values were in line with those described above for the other set of materials. Textural parameters for MCM materials (Table 2) were also found to match values reported in the literature for related systems [24].

**Figure 5:**
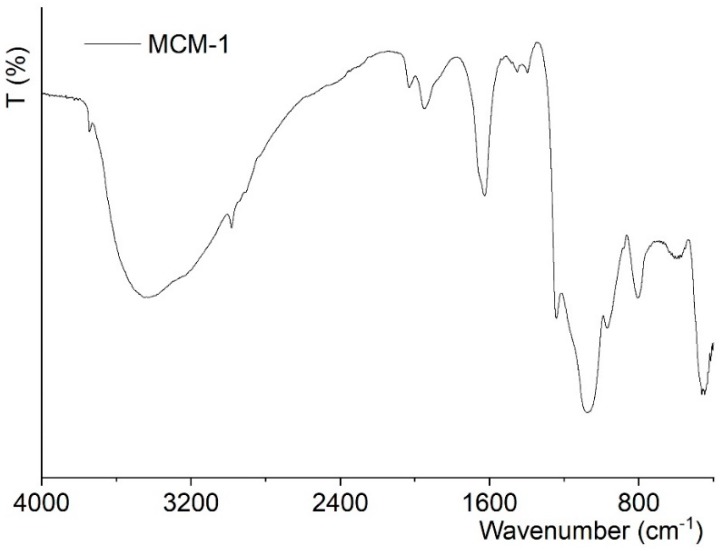
Infrared spectrum of the material MCM-1.

The DRIFT spectrum (Figure 5) of the parent MCM shows a broad band at 3600–3000 cm^−1^, which can be assigned to the O–H stretching vibrations of hydrogen bonded silanol groups. Other important features comprised the band at ca. 1634 cm^−1^ due to OH bending modes and an intense broad band at 1240–950 cm^−1^ associated with asymmetric stretching vibration modes of the mesoporous framework (νSi–O–Si) [25].

The introduction of the IMP-SI ligand to obtain the functionalized material MCM-IMP-Si did not significantly change the absorption profile of the mesoporous host material, but new bands characteristic of the ligand present inside the pores were now detected. Grafting of the IMP ligand was monitored by probing its νC=N mode, observed in the pyridine moiety of the free IMP ligand at 1655 cm^−1^. After grafting, this mode was blue shifted to 1652 cm^−1^, as expected after the formation of silyl esters and documented in the literature [26]. After binding the molybdenum complex [MoBr(η^3^-C_3_H_5_)(CO)_2_(MeCN)_2_] to afford material MCM-1, two new bands corresponding to the νC≡O stretching modes were observed in the DRIFT spectra, at 2031 and 1934 cm^−1^, having shifted from 1942 and 1854 cm^−1^ in **1**.

Additionally, the absence of the bands due to the νC≡N vibrational modes from the acetonitrile (MeCN) ligands showed that they had been replaced by the immobilized ligand. Similar results were obtained for materials with ligands Me-IMP-Si and Ph-IMP-Si, after the functionalization with the Mo(II) core, as well as for the new materials MCM-2 and MCM-3.

**Figure 6:**
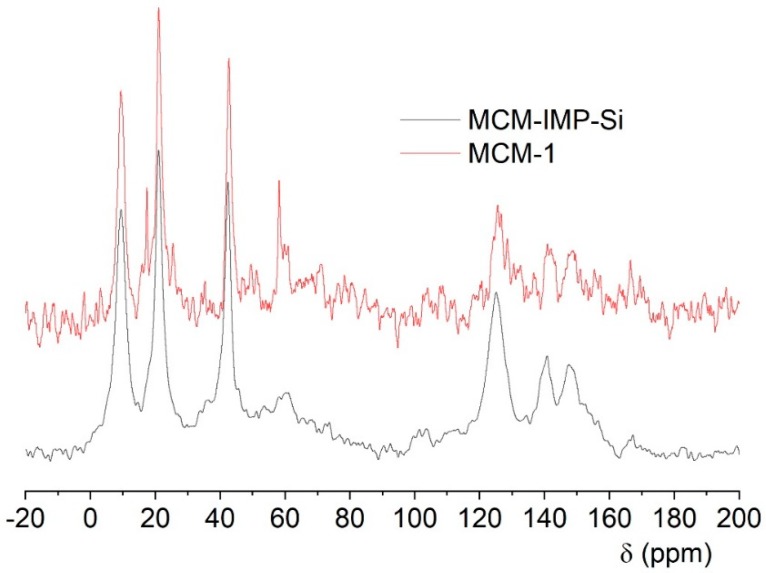
^13^C CP MAS NMR spectra of materials MCM-IMP-Si and MCM-1.

^13^C and ^29^Si solid-state NMR spectroscopy studies were also performed on the materials and the resonances observed were assigned according to the literature [26]. ^13^C CP MAS solid-state NMR spectra for MCM-IMP-Si and MCM-1 are presented in Figure 6. ^13^C CP MAS solid-state NMR spectra for MCM-Me-IMP-Si, MCM-2, MCM-Ph-IMP-Si, and MCM-3 are not presented, nevertheless the results were similar to those obtained for the previous ones and were in accordance with results described in the literature.

**Figure 7:**
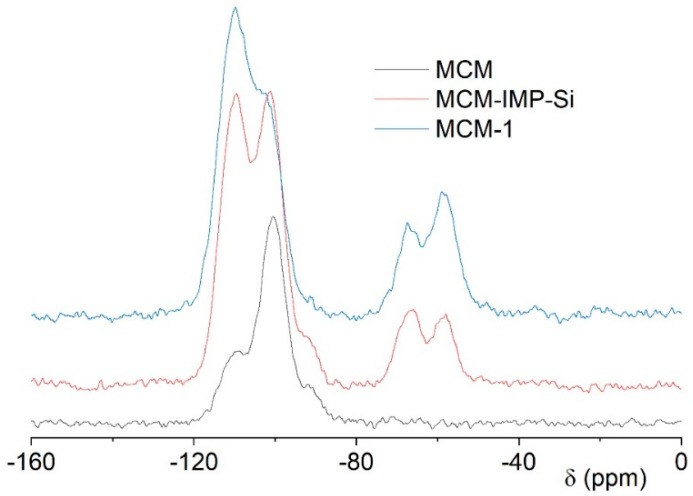
^29^Si CP MAS NMR spectra for the materials MCM, MCM-IMP-Si, and MCM-1.

In the ^13^C solid-state spectra of the IMP-Si ligand (Figure 6), three strong signals were observed in the 0–100 ppm range, corresponding to the aliphatic carbons closer to the SiOR group of IMP-Si. The signal at the lower δ was attributed to the carbon linked to Si, at 9.7 ppm, while those at 21.2 and 42.6 ppm were assigned to the other two methylene carbon atoms of the aliphatic chain. Three signals observed at 125.6, 141.2, and 148.1 ppm correspond to the aromatic carbon atoms of the IMP-Si ligand. In general, the same signals were observed in the ^13^C solid-state NMR spectra of the material after the reaction of the Mo(II) core with precursor materials. It was possible to assign the signals at 10.8, 22.3, and 44.0 ppm to the aliphatic part of the ligand, and those at 126.6, 141.8, and 149.1 ppm to the aromatic part of the ligand, in MCM-1. No carbon signals from the carbonyls of this material could be observed. Figure 7 shows the ^29^Si CP MAS NMR spectra for pristine MCM and the derivatized MCM-IMP-Si and MCM-1 materials. The parent MCM material displayed three broad convoluted resonances in the ^29^Si CP MAS NMR spectrum at −99.9 and −109.8ppm, assigned to Q^3^ and Q^4^ species of the silica framework, respectively [Q^n^ = –Si(OSi)_n_(OH)_4−n_]. A weak shoulder was also observed at −90.4 ppm, due to the Q^2^ species. The ^29^Si CP MAS spectrum of MCM-IMP-Si material also displayed two broad signals at −101.0 and −109.0 ppm, assigned to Q^3^ and Q^4^ organosilicon species, respectively, and it was also possible to observe a small shoulder at −92.1 assigned to the Q^2^ species. New signals corresponding to the T^1^, T^2^, and T^3^ species of the Si(OR)_3_ groups of the IMP-Si ligand were also observed at −49.7, −57.8, and −65.4 ppm, respectively.

The ^29^Si CP MAS spectra in Figure 7 show that reaction of MCM-IMP-Si with the organometallic core complex Mo(II) did not significantly change the environment of Si, as expected, indicating that the metal fragment reacted with the immobilized ligand and did not interact with the wall surface. It was possible to assign signals at −106.5, −107.8, −102.6, −67.0, −58.8, and −47.8 ppm to the Q^2^, Q^3^, Q^4^, T^1^, T^2^ and T^3^ species, respectively.

The previously described characterization techniques indicated that the surface of the MCM porous material was functionalized with the three ligands, IMP-Si, Me-IMP-Si, and Ph-IMP-Si, which in turn reacted with the Mo(II) metal fragments to yield metal-containing materials.

### 2.3. Catalytic Studies

The catalytic activity of complexes **1**, **2**, and **3** (Table 3) and materials MCM-1, MCM-2, and MCM-3 (Table 4) was studied in the olefin epoxidation reaction with *tert*-butyl hydroperoxide (tbhp) as the oxidant. Different substrates were chosen in order to gain insight on selectivity, namely stereoselectivity: *cis*-cyclooctene (cy8), styrene (sty), *cis*-hex-3-en-1-ol (*cis*), *trans*-hex-3-en-1-ol (*trans*), geraniol (ger), 1-octene (1-oct), and *S*-(+)-limonene (S-lim). No conversion of any substrate was observed in blank runs without a catalyst and in the presence of an oxidizing agent.

All of the complexes were able to oxidize cy8 and cis with 99–100% conversion, and selectivity for the respective epoxide and yield (entries 1–3, 7–9), as had been observed for many related Mo(II) complex precursors. Very high conversions (98%) were determined for ger, but two products were formed in ~1:1 ration, the epoxide (2,3-oxyrane) and 6,7-oxyrane (entries 13–15). Relatively high conversions (90 and 82%) with **1** and **2**, and medium (50%) with **3** were found in the oxidation of trans (entries 10–12). In all cases, the selectivity for the epoxide was 86–89%, but a second product (aldehyde) was also formed. 1-oct was not easily oxidized, and conversions reached 55, 43, and 72% with **1**, **2**, and **3**, respectively, with epoxide selectivity in the range of 96–100% (entries 16–18). The second by-product (1-oct-2-ketone) was formed in very small amounts. Sty conversions did not go beyond 40%, and two products, the epoxide and benzaldehyde, were formed in ~3:2 ratios (entries 4–6). The differences between the activity of the three complexes were very small, suggesting that the change in substituent (H, Me, and Ph) had negligible effect. Only for sty was complex **1** less active than the others. The complexes oxidized cis more efficiently than trans, with higher conversion and selectivity for the epoxide, although the differences were small. On the other hand, we showed in a recent work [26] that, for a series of α-diimine (derivatives of 2,2′-bipyridyl and 1,10-phenantroline) complexes of MoBr(η^3^-C_3_H_5_)(CO)_2_, the selectivities were higher for *cis* but the conversions were lower. These ligands were more symmetrical than Y-IMP, and the approach of the trans substrate to the active species may have been more restricted than that of *cis*.

The immobilization of the three complexes in MCM did not improve their capability as catalysts. This result was surprising, since in a previous work dealing with another Mo(II) catalyst precursor with the same ligand ([MoX_2_(CO)_3_(IMP)]), activity increased when the metal complex was supported in MCM [26]. However, the allylic Mo(II) system described in the present work was usually a better catalyst [27].

Epoxidation of cy8 remained very efficient when any of the three materials acted as catalysts (entries 1–3), but conversions of *cis* into its epoxide became lower, even though the selectivity was kept (entries 4–6). The conversions of ger (entries 13–15) decreased from 98% to 56, 66, and 23% for MCM-1, MCM-2, and MCM-3, respectively, and the relative amount of the 2,3-oxyrane also was tendentially lower for the two more active catalysts. The oxidation of *trans* was interesting, in the sense that no epoxide was formed and the reaction was 100% selective for the aldehyde (entries 10–12), with conversions slightly higher than in the homogeneous catalysts **1** and **2**, and significantly higher for **3**. The heterogeneous catalysts, except MCM-2, were less active than the homogeneous ones in the oxidation of 1-oct, and the selectivity decreased to very modest values (less than half, entries 16–18). On the other hand, higher conversions took place for the three catalysts with sty, and the selectivity toward the epoxide increased from ~2:1 to ~3:1(entries 4–6), with MCM-3 behaving in a different way than the others (much less epoxide formed). Another substrate, S-lim, was tested with the materials as catalysts. The conversions were high, in the range of 83–89% (entries 19–21), but the yield of epoxide was very low, even considering both Z- and E-epoxides. The E- and Z-alcohols were obtained in much higher proportion. The selectivity toward the epoxide was extremely low.

The analogous complex with 8-aminoquinoline [27], for instance, displayed a much lower conversion in the oxidation of *cis* upon immobilization (14 compared with 69%), but the opposite was observed with trans (56 and 75%), with the selectivity always at 100%. The lower activity of the heterogeneous catalysts in general may be explained by the steric hindrance of the side chain of the Y-IMP complexes, which seemed to be more effective in the confined pores of the material.

The catalytic performance of the heterogeneous systems was also assessed in terms of leaching and recycling. To study the leaching, a catalytic experiment, using cy8 and MCM-1, was started and the catalyst was filtered from the mixture after 2 h. The conversion at this point was 82% and after 24 h only 86% (Figure S6), while it reached 100% in the reference experiment with the catalyst present the entire time. This showed that the reaction practically stopped when the catalyst was removed, indicating a small amount of leaching to the homogeneous phase. Therefore, the material acted as a heterogeneous catalyst. The recycling experiments were performed for the same system (cy8 and MCM-1). After one 24 h cycle, the solid catalyst was separated; another load of substrate, solvent, and oxidant was added; and the reaction was followed for another 24 h. The conversions were 100%, 79%, and 60% in the first, second, and third cycle, respectively, reflecting a loss of activity of ~20% between cycles.

The Mo(II) complexes and the Mo(II)-containing materials were catalyst precursors that had to be oxidized to Mo(VI) by tbhp in the first step to afford the catalysis active species. In this process, the allyl and the carbonyl ligands were lost and the Mo=O and Mo-O-Mo bonds were formed, with the bidentate ligand—here Y-IMP—remaining bound to the metal [27]. The complexes **1**–**3** were fluxional in solution, as reflected in their NMR spectra, and, more surprisingly, the two main isomers even coexisted in the solid for **1**. This suggested that several isomers of the active species (see Scheme 3) were more likely to exist in the solution than with sterically crowded ligands, such as 2,9-R_2_-1,10-phenanthroline (R = Me, tBu) [27]. This could be a possible explanation for the lack of selectivity observed in general. The immobilization procedure may have also ended up with several supported species, which would then be oxidized.

### 2.4. Computational Studies

Density functional theory (DFT) calculations [28] were performed using the Amsterdam Density Functional (ADF) program [29] (more details in Experimental) in order to explore the energy difference between the axial and the equatorial isomers. As described above, for the IMP ligand, the two isomers could coexist not only in solution but also in the solid state, which was an unprecedented finding. Indeed, the Me-IMP complex was observed in the solid state as the axial isomers. It was found that the axial isomer was the most stable for the three complexes by small amounts, namely 1.20 for **1**, 1.38 for **2**, and 1.13 kcal·mol^−1^ for **3**. The meaning of these values is that the two isomers had essentially the same energy and the crystal packing had a determining effect on the species present in the solid state. A view of relevant packing motifs for **1** and **2** can be seen in Figure S7 (SI). A slight change to the methodology, for instance by optimizing without considering the solvent, led to the equatorial isomer of **1** being more stable by only 0.13 kcal·mol^−1^. A close look at the molecular structures (Figure S7 in SI) suggested that the addition of the Me or Ph substituent would influence the solid-state structure.

## 3. Materials and Methods

All reagents were obtained from Aldrich and used as received. All the work involving sensitive compounds was carried out using standard Schlenk techniques. Commercial-grade solvents were dried and deoxygenated by standard procedures (i.e., THF, toluene, and benzene over Na/benzophenone ketyl, CH_2_Cl_2_ over CaH_2_), distilled under nitrogen, and kept over 4 Å molecular sieves. The organometallic complex [Mo(η^3^-C_3_H_5_)Br(CO)_2_(CH_3_CN)_2_] [30,31] and the ligands L1–L3 were prepared as previously described [32]. Ligands C_5_H_4_NCY=N(CH_2_)_2_CH_3_ (Y = H, L2 Y = CH_3_, L3 Y = Ph, L4) and C_5_H_4_NCY=N(CH_2_)_3_Si(OEt_3_) (Y = H, L2Si Y = CH_3_, L3Si) were prepared as reported [32]. MCM-41 and post synthetic derivatized materials MCM-IMP-Si, MCM-Me-IMP-Si, and MCM-Ph-IMP-Si were synthesized as previously described, using [(C_16_H_33_)N(CH_3_)_3_]Br (CTAB) as the templating agent [32]. Before the grafting experiment, physisorbed water was removed from calcined (540 ᵒC for 6 h under air) MCM-41 by heating at 180 °C in vacuum (10^−2^ Pa) for 2 h. Moreover, hybrid materials were prepared using ligands 3-chloropropyltriethoxysilane, L2Si, and L3Si as reported, with an organic material load of ≈3% [11,33,34].

FTIR spectra were obtained on a Nicolet 6700 in the 400–4000 cm^−1^ range with 4 cm^−1^ resolution, using KBr pellets for complexes and DRIFT for materials. Powder XRD measurements were recorded with a Philips Analytical PW 3050/60 X’Pert PRO (θ/2θ) equipped with X’Celerator detector and with automatic data acquisition (X’Pert Data Collector (v2.0b) software, Philips, Eindhoven, Netherlands), using a monochromatic Cu Kα radiation as the incident beam, operating at 40 kV–30 mA. XRD diffraction patterns were obtained by continuous scanning in a 2*θ*-range of 2 to 10° with 2*θ*-step size of 0.017° and a scan step time of 99.695 s. A Bruker Avance 400 spectrometer with frequencies of 400.13 MHz for ^1^H and 100.61 MHz for ^13^C was used to obtain ^1^H and ^13^C solution NMR spectra. The solid-state NMR spectra were obtained on a (9.4 T) Bruker Avance 400P spectrometer at the University of Aveiro by Dr. Paula Ferreira (Bruker, Billerica, MA, USA). The ^29^Si spectra were recorded with a frequency of 79.49 MHz. ^29^Si MAS NMR spectra were recorded with 40° pulses, spinning rates of 5.0–5.5 kHz, and 60 s recycle delays and ^29^Si CP MAS NMR spectra with 5.5 μs 1H 90° pulse, 2 ms contact time, a spinning rate of 8 kHz, and 4 s recycle delays. Chemical shifts are given in ppm with tetramethylsilane (TMS) as reference. ^13^C solid-state NMR spectra were recorded at 125.76 MHz on a Bruker Avance 500 spectrometer (Bruker, Billerica, MA, USA). The N_2_ sorption was measured in an automatic apparatus (ASAP 2010; Micrometrics). BET specific surface areas (*S_BET_*, *p*/*p*_0_ from 0.03 to 0.13) and specific total pore volume, *V_p_*, were estimated from N_2_ adsorption isotherms measured at 77 K. The PSD were calculated by the BJH method using the modified Kelvin equation, with correction for the statistical film thickness on the pore walls [24]. The statistical film thickness was calculated using the Harkins-Jura equation in the *p*/*p*_0_ range from 0.1 to 0.95. Microanalyses (C, N, S, H) and atomic absorption for determination of molybdenum and tungsten were performed at the Institute of Chemical and Biological Technology (Instituto de Tecnologia Química e Biológica, or ITQB) and the University of Vigo.

### 3.1. Synthesis of [MoBr(η^3^-C_3_H_5_)(CO)_2_C_5_H_4_NCH=N(CH_2_)_2_CH_3_] (Complex **1**)

A solution of [MoBr(η^3^-C_3_H_5_)(CO)_2_(CH_3_CN)_2_] (1 mmol) in dichloromethane (10 mL) was added to a solution of the ligand C_5_H_4_NCH=N(CH_2_)_2_CH_3_ (1.2 mmol, 0.177 g), which was also in dichloromethane (10 mL). The resulting mixture was allowed to stir for 6 h at room temperature and under a nitrogen atmosphere. The solution was then filtered and concentrated by evaporation. After the addition of pentane, a purple crystalline powder precipitated. The product was filtered and dried under a vacuum.

Yield (η): 88 % (0.36 g).

Elemental analysis MoBrC_14_H_17_N_2_O_2_ (421.14)%: Calc C 39.93, N 6.65, H 4.07; found C 37.21, N 6.29, H 4.05.

IR (KBr pellets, ν cm^−1^): 3075 (w), 3044 (m), 2997 (m), 2967 (m), 2936 (w), 2870 (m), 1942 (s), 1845 (vs), 1654 (m), 1647 (m), 1599 (m), 1470 (m), 1306 (w), 1262 (s), 1251 (w), 1152 (w), 1103 (s), 1024 (s), 978 (m), 803 (s), 795 (s), 668 (w), 628 (w), 572 (m).

^1^H-NMR (400.13 MHz, (*CD*_3_)_2_(CO), room temp., ppm): 1.09 (t, 3H, H_1_), 1.25, 1.44 (H_anti_), 2.31 (t, 2H, H_2_), 2.95–4.00 (H_syn_), 4.24 (t, 2H, H_3_), 4.46 (H_meso_), 7.48 (t, 1H, H_8_), 7.69 (d, 1H, H_6_), 7.86 (t, 1H, H_7_), 8.24 (s, 1H, H_4_), 8.48 (d, 1H, H_9_).

^13^C{1H} NMR (100.61 MHz, (*CD*_3_)_2_(CO), room temp., ppm): 11.6 (C1), 23.5 (C2), 55.1-68.4 (C_allyl_), 72.7 (C3), 126.3 (C6), 127.6 (C8), 137.7 (C7), 150.2 (C9), 152.7 (C5), 160.2 (C4).

### 3.2. Synthesis of [MoBr(η^3^-C_3_H_5_)(CO)_2_C_5_H_4_NCCH_3_=N(CH_2_)_2_CH_3_] (Complex **2**)

A solution of [MoBr(η^3^-C_3_H_5_)(CO)_2_(CH_3_CN)_2_] (0.5 mmol) in dichloromethane (10 mL) was added to a solution of the ligand C_5_H_4_NCCH_3_=N(CH_2_)_2_CH_3_ (1.2 mmol, 0.194 g), which was also in dichloromethane (10 mL). The resulting mixture was allowed to stir for 6 h at room temperature and under a nitrogen atmosphere. The solution was then filtered and concentrated by evaporation, yielding a pink crystalline powder. The product was filtered and recrystallized with hexane, and dried under a vacuum.

Yield (η): 90 % (0.39 g).

Elemental analysis MoBrC_15_H_19_N_2_O_2_ (435.17)%: Calc C 41.4, N 6.44, H 4.4; found C 41.15, N 6.27, H 3.95.

IR (KBr pellets, ν cm^−1^): 3072 (m), 3047 (w), 3021 (w), 2968 (m), 2933 (m), 2873 (w), 1936 (s), 1853 (vs), 1593 (m), 1562 (m), 1435 (m), 1375 (w), 1323 (m), 1302 (s), 1254 (w), 1051 (w), 1026 (s), 928 (m), 793 (s), 754 (s), 658 (w), 633 (w), 560 (m).

^1^H-NMR (400.13 MHz, C*D*Cl_3_, room temp., ppm): 1.24 (t, 3H, H_1_), 1.49, 1.61 (H_anti_), 2.25 (m, 2H, H_2_), 2.58 (s, 3H, H_10_), 3.88 (t, 2H, H_3_), 4.11, 4.26 (H_syn_), 4.41, 4.80 (H_meso_), 7.53 (t, 1H, H_8_), 7.87 (d, 1H, H_6_), 7.94 (t, 1H, H_7_), 8.47 (d, 1H, H_9_).

^13^C{1H} NMR (100.61 MHz, C*D*Cl_3_, room temp., ppm): 11.9 (C1), 16.2 (C2), 22.9 (C10), 55.2, 61.5 (C_allyl-terminal_), 64.0 (C_allyl-center_), 72.8 (C3), 125.6 (C7), 126.2 (C6), 137.6 (C8), 150.1 (C9), 154.6 (C5), 165.7 (C4).

### 3.3. Synthesis of [MoBr(η^3^-C_3_H_5_)(CO)_2_C_5_H_4_NCPh=N(CH_2_)_2_CH_3_] (Complex **3**)

A solution of [MoBr(η^3^-C_3_H_5_)(CO)_2_(CH_3_CN)_2_] (0.5 mmol) in dichloromethane (10 mL) was added to a solution of the ligand C_5_H_4_NCCH_3_=N(CH_2_)_2_CH_3_ (1.2 mmol, 0.269 g), which was also in dichloromethane (10 mL). The resulting mixture was allowed to stir for 6 h at room temperature and under a nitrogen atmosphere. The solution was then filtered and concentrated by evaporation, yielding a violet crystalline powder. The product was filtered and recrystallized with hexane, and dried under a vacuum.

Yield (η): 85 % (0.41 g).

Elemental analysis MoBrC_19_H_21_N_2_O_2_ (485.23)%: Calc C 47.03, N 5.77, H 4.36 found C 46.72, N 5.49, H 4.01.

IR (KBr pellets, ν cm^−1^) 3106 (w), 3058 (m), 2977 (m), 2929 (m), 2874 (m), 1924 (vs), 1848 (vs), 1586 (s), 1554 (m), 1468 (s), 1443 (s), 1325 (s), 1257 (m), 1164 (w), 1017 (s), 953 (w), 801 (s), 756 (s), 705 (s).

^1^H-NMR (400.13 MHz, (*CD*_3_)_2_(CO), room temp., ppm): 0.73, 0.97 (2t, 6H, H_1_), 1.16, 1.25, 1.34, 1.48 (H_anti_), 2.29, 2.45 (2m, 4H, H_2_), 3.14, 3.28 (H_syn_), 3.58, 3.76, 3.95 (H_meso_), 4.08 (m, 2H, H_3_), 7.20 (t, 1H, H_12_), 7.20 (d, 1H, H_14_), 7.52 (m, 2H, H_11_ H_15_), 7.65 (t, 1H, H_8_), 7.72 (t, 1H, H_13_), 8.01 (t, 1H, H_7_), 8.74 (d, 1H, H_6_), 8.91 (d, 1H, H_9_).

^13^C{1H} NMR (100.61 MHz, (*CD*_3_)_2_(CO), room temp., ppm): 11.0 (C1), 23.2 (C2), 52.7, 53.2, 63.5 (C_anti_), 52.9, 53.0, 59.6 (C_syn_), 62.6, 73.3 (C_meso_), 72.0 (C3), 126.6 (C11), 127.7 (C12), 128.9 (C14 C15), 129.0 (C8), 130.9 (C13), 138.2 (C7), 150.3 (C6), 152.2 (C9), 155.3 (C4).

### 3.4. Synthesis of Materials MCM-IMP-Si, MCM-Me-IMP-Si, and MCM-Ph-IMP-Si

#### MCM-Ph-IMP-Si

A suspension of MCM (0.8 g) in toluene (10 mL) was added to a solution of pycaPhSi (1.13 mmol, 0.437 g) in toluene (10 mL) and was heated at 375 K for 9 h with stirring. The resulting yellowish solid was filtered, washed four times with dichloromethane (4 × 15 mL), and dried in a vacuum for 3 h at 323 K.

IV (KBr, ν cm^−1^) 3402 (vs), 3213 (s), 2920 (s), 2006 (w), 1626 (s), 1618 (s), 1541 (m), 1466 (w), 1446 (w), 1398 (m), 1309 (m), 1221 (s), 1051 (vs), 953 (s), 793 (m), 696 (m), 559 (w), 463 (m).

### 3.5. Synthesis of Materials MCM-1, MCM-2, and MCM-3

A solution of [MoBr(η^3^-C_3_H_5_)(CO)_2_(CH_3_CN)_2_] (0.65 mmol) in dry toluene (10 mL) was added to a suspension of 1 g of the material MCM-IMP-Si, MCM-Me-IMP-Si, or MCM-Ph-IMP-Si, in dry toluene (20 mL). The reaction mixture was stirred under a N_2_ atmosphere at reflux temperature overnight. The resulting material was then filtered off, washed with CH_2_Cl_2_, and dried under a vacuum for 3 h.

**MCM-[MoBr(η^3^-C_3_H_5_)(CO)_2_(IMP)] (MCM-1)** (pink powder). Elemental analysis (%) found C 6.01, N 1.64, H 1.92, Mo 4.9.

IR (KBr ν cm^−1^) 2983 (w), 2947 (w), 2902 (w; ν_N-H_), 2031, 1934 (m; ν_C≡O_), 1618 (s; ν_C=N_), 1446 (m), 1396 (m), 1234 (vs), 1061 (vs), 957 (vs), 796 (s).

^13^C CPMAS NMR (δ ppm) 9.0 (SiCH_2_), 17.45 (SiOCH_2_CH_3_), 21.33 (CH_2_CH_2_CH_2_), 42.97 (CH_2_N), 58.12 (SiOCH_2_CH_3_)

^29^Si CPMAS NMR (δ ppm) -58.92 (T^2^), −67.49 (T^3^), −102.34 (Q^3^), −109.80 (Q^4^) ^29^Si MAS NMR (δ ppm) −58.13 (T^2^), −68.84 (T^3^), −103.35 (Q^3^), −108.81 (Q^4^).

**MCM-[MoBr(η^3^-C_3_H_5_)(CO)_2_(Me-IMP)]** (**MCM-2**) Elemental analysis (%) found C 5.08, N 2.03, H 1.90, Mo 4.79.

IR (KBr ν cm^−1^) 3066 (w), 3032 (w), 2976 (m), 2924 (w) 1938, 1833, (s; ν_C≡O_), 1653, 1618 (vs; ν_C=N_), 1495 (m), 1448 (m), 1388 (m), 1240 (vs), 1105 (vs), 798 (vs), 735 (s).

^13^C CPMAS NMR (δ ppm) 9.53 (SiCH_2_), 17.64 (SiOCH_2_CH_3_), 20.94 (CH_2_CH_2_CH_2_), 42.25 (CH_2_N), 58.26 (SiOCH_2_CH_3_).

^29^Si CPMAS NMR (δ ppm) −49.83 (T^1^), −58.62 (T^2^), −66.30 (T^3^), −102.25 (Q^3^), −109.85 (Q^4^) ^29^Si MAS NMR (δ ppm) −55.44 (T^2^), −65.51 (T^3^), −102.41 (Q^3^), −108.04 (Q^4^).

**MCM-[MoBr(η^3^-C_3_H_5_)(CO)_2_(Ph-IMP)]** (**MCM-3**) Elemental analysis (%) found C 14.16, N 2.68, H 2.26, Mo 3.45.

IV (KBr, ν cm^−1^) 3415 (vs), 2960 (s), 2927 (s), 1936 (m), 1844 (m), 1621 (m), 1493 (vw), 1321 (w), 1229 (vs), 1061 (vs), 942 (m), 791 (m), 691 (w), 550 (w), 449 (s).

Single crystal X-ray diffraction data of **1** and **2** were collected using a Bruker SMART APEX II diffractometer (Bruker, Billerica, MA, USA) equipped with a CCD area detector and graphite-monochromated Mo-Kα radiation (λ = 0.71073 Å). Data were corrected for absorption, as well as for Lorentz and polarization effects. The structures were solved by intrinsic phasing with the SHELXT program [35] and refined by full-matrix least-squares using the SHELXL program [36]. All non-hydrogen atoms were refined with anisotropic thermal displacements. The hydrogen atoms were positioned in geometrically idealized positions and refined with isotropic temperature factors depending on the parent atom using a ridging model. The exception was the positions of the terminal hydrogen atoms (two CH_2_ groups) of the allyl ligand, which were obtained from last difference Fourier maps calculated for each complex, and subsequently refined with individual isotropic temperature factors. Crystal data and selected refinement details are summarized in Table S2. Solid-state structures were drawn with the Mercury software [37]. Crystal structures were deposited with Cambridge Crystallographic Data Centre (CCDC) under the following CCDC numbers: 1888029 (**1**) and 1888030 (**2),** and can be obtained free of charge via http://www.ccdc.cam.ac.uk/data request/cif.

DFT calculations [38] were performed using the ADF program package [38]. The geometries were optimized with gradient correction, using the Local Density Approximation of the correlation energy (Vosko-Wilk-Nusair) [38], and the Generalized Gradient Approximation (Becke’s [39,40] exchange and Perdew’s [41] correlation functionals), without symmetry constraints. Relativistic effects were accounted with the ZORA approximation [42]. The core orbitals were frozen for O, C, and N (1s) and S (1s, 2s, 2p). Triple ζ Slater-type orbitals (STO) were used to describe the valence shells O, C, and N (2s and 2p). A set of two polarization functions was added to O, C, and N, (single ζ, 3d, 4f). Triple ζ STO were used to describe the valence shells of H (1s) augmented with two polarization functions (single ζ 2s, 2p). The structures were modeled after those of compounds **1** and **2** described above. Three-dimensional structures were obtained with Chemcraft [43].

## 4. Conclusions

The structure of the new complex [Mo(η^3^-C_3_H_5_)Br(CO)_2_{^i^PrN=C(H)C_5_H_4_N}], where ^i^PrN=C(H)C_5_H_4_N=IMP = N-isopropyl 2-iminomethylpyridine), was particularly interesting since the two isomers of this seven-coordinate and pseudo-octahedral complex were for the first time found [44] in the crystal, and there was no particular disorder. The Me analogue, [Mo(η^3^-C_3_H_5_)Br(CO)_2_{^i^PrN=C(Me)C_5_H_4_N}] crystallized as the less common axial isomer, essentially observed in Mo(η^3^-C_3_H_5_)X(CO)_2_ complexes of bis(4-Y-phenyl)-acenaphthenequinone diimine (X = Cl, Br, SCN; Y can be a variety of substituents), owing to their п-acceptor capability. In all cases analyzed, the DFT calculated energy difference was very small and the packing forces determined the outcome.

The complexes were immobilized in MCM-41, and their activity as olefin epoxidation catalysts remained or decreased slightly when going from the homogeneous to the heterogeneous catalyst.

## Figures and Tables

**Table 1:** Selected bond distances (Å) and angles (°) for the two independent molecules of [Mo(η^3^-C_3_H_5_)Br(CO)_2_(IMP)] (**1**) and for [Mo(η^3^-C_3_H_5_)Br(CO)_2_(Me-IMP)] (**2**).

Complex Isomer	1 (Equatorial)	1 (Axial)	2
Bonds			
Mo-C(1)	2.2995(16)	2.3266(16)	2.3442(14)
Mo-C(2)	2.2072(15)	2.2136(14)	2.2148(14)
Mo-C(3)	2.3273(16)	2.3182(16)	2.3163(15)
Mo-Br	2.6961(2)	2.6408(2)	2.71031(19)
Mo-N(11)	2.2833(12)	2.2371(12)	2.2652(12)
Mo-N(18)	2.2031(12)	2.2636(12)	2.2049(11)
Mo-C(4)	1.9398(16)	1.9680(16)	1.9561(15)
Mo-C(6)	1.9695(16)	1.9541(16)	1.9468(15)
N(11)-Mo-N(18)	73.36(4)	72.74(4)	71.89(4)
N(11)-Mo-Br	81.22(3)	81.49(3)	85.15(3)
Angles			
N(18)-Mo-Br	83.46(3)	82.59(3)	80.67(3)
C(4)-Mo-C(6)	80.84(7)	78.35(6)	76.26(6)
C(4)-Mo-Br	86.62(5)	168.52(5)	166.23(4)
C(6)-Mo-Br	89.00(5)	96.79(4)	96.86(4)
C(4)-Mo-N(11)	167.69(5)	100.68(5)	98.56(5)
C(4)-Mo-N(18)	103.47(6)	87.28(6)	87.86(5)
C(6)-Mo-N(11)	100.74(6)	166.00(5)	165.34(5)
C(6)-Mo-N(18)	171.03(6)	93.26(5)	94.04(5)

**Table 2:** Textural parameters of host and composite materials from powder XRD and N_2_ isotherms at 77 K, for all prepared materials.

Materials	*d*_100_ (Å)	*a* (Å)	*S_BET_*/m^2^·g^−1^	Δ*S_BET_*/%	*V_p_*/cm^3^·g^−1^	Δ*V_p_* ^b^/%	*d_BJH_*/(Å)
MCM	35.6	41.1	1037	-	0.97	-	36.0
MCM-IMP-Si	36.5	42.1	558	−46	0.42	−57	32.6
MCM-1	36.2	41.8	553	−47	0.36	−63	28.5
MCM	35.6	41.1	1037	-	0.97	-	36.0
MCM-Me-IMP-Si	36.2	41.8	674	−35	0.42	−57	28.7
MCM-2	36.0	41.6	523	−50	0.25	−74	26.7
MCM	36.0	41.6	1031	-	0.88	-	32.8
MCM-Ph-IMP-Si	36.7	42.4	683	−33.8	0.46	−48	28.5
MCM-3	37.2	42.9	547	−47.0	0.31	−65	26.6

**Table 3:** Olefin epoxidation of *cis*-cyclooctene (cy8), styrene (sty), *cis*-hex-3-en-1-ol (*cis*), *trans*-hex-3-en-1-ol (*trans*), geraniol (ger), and 1-octene (1-oct) catalyzed by complexes **1**–**3**.

Entry	Reaction ^a^	Catalyst	Conv. % ^b^	Select.% ^c^	Yield %
1	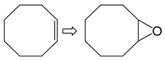	1	99	100	99
2		2	99	100	99
3		3	100	100	100
4	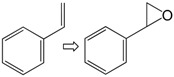	1	29	58	17/12 ^d^
5		2	33	41	20/13 ^d^
6		3	40	36	26/14 ^d^
7	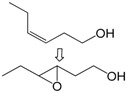	1	99	100	99
8		2	99	100	99
9		3	99	100	99
10	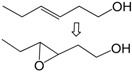	1	90	89	80/10 ^e^
11		2	82	86	70/12 ^e^
12		3	50	89	45/5 ^e^
13	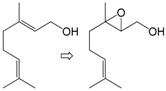	1	98	49	50/48 ^f^
14		2	98	50	49/49 ^f^
15		3	98	49	50/48 ^f^
16	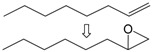	1	55	98	54/1 ^g^
17		2	43	96	41/2 ^g^
18		3	72	100	72

^a^ All reactions were carried out in dichloromethane in the presence of 2 eq. of oxidant tert-Butyl hydroperoxide (tbhp) and 175 mg of catalyst at 328 K; ^b^ calculated after 24 h; ^c^ calculated as “Yield of epoxide”/“Conversion” × 100%; ^d^ benzaldehyde as by-product; ^e^ aldehyde as by-product; ^f^ Yield of 6,7-oxyrane product; and ^g^ yield of 1-oct-2-ketone product.

**Table 4:** Olefin epoxidation of *cis*-cyclooctene (cy8), styrene (sty), *cis*-hex-3-en-1-ol (*cis*), *trans*-hex-3-en-1-ol (*trans*), geraniol (ger), 1-octene (1-oct), and *S*-(+)-limonene (*S*-lim) catalyzed by materials MCM-1, MCM-2, and MCM-3.

Entry	Reaction ^a^	Catalyst	Conv. % ^b^	Select. % ^c^	Yield %
1	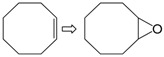	MCM-1	100/79/60	100/100/100	100
2		MCM-2	100	100	100
3		MCM-3	100	100	100
4	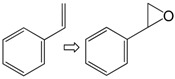	MCM-1	40	82	33/7 ^d^
5		MCM-2	45	80	36/9 ^d^
6		MCM-3	85	12	10/75
7	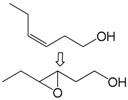	MCM-1	73	100	73
8		MCM-2	76	100	76
9		MCM-3	53	100	53
10	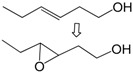	MCM-1	75	0	0 ^e^
11		MCM-2	80	0	0 ^e^
12		MCM-3	75	0	0 ^e^
13	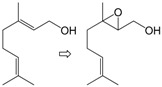	MCM-1	56	59	23/33 ^f^
14		MCM-2	66	58	28/38 ^f^
15		MCM-3	23	50	12/11 ^f^
16	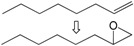	MCM-1	35	38	13/22 ^h^
17		MCM-2	42	45	19/23 ^h^
18		MCM-3	14	34	5/9 ^h^
19	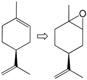	MCM-1	83	23	3/16/30/34 ^g^
20		MCM-2	87	24	4/17/30/36 ^g^
21		MCM-3	89	37	7/26/28/28 ^g^

^a^ All reactions were carried in dichloromethane in the presence of 2 eq. of oxidant (tbhp) and 175 mg of catalyst at 328 K; ^b^ calculated after 24 h; ^c^ calculated as “Yield of epoxide”/“Conversion” × 100%; ^d^ benzaldehyde as by-product; ^e^ aldehyde as by-product; ^f^ yield of 6,7-oxyrane product; ^g^ yield of Z-epoxide, E-epoxide, Z-limonene alcohol, and E-limonene alcohol products; and ^h^ yield of 1-oct-2-ketone product.

## Supplementary Materials

The following are available online, Figure S1: NMR spectra of **1**: ^1^H (bottom) and ^13^C (top), Figure S2: ^1^H-NMR spectra of ligand Me-IMP (center), complex **2** (top), and ^13^C-NMR spectrum of complex **2**, Figure S3: Selection of the upfield region of the bidimensional COSY spectrum of complex **3**, Figure S4: Selection of the upfield region of the HMQC spectrum of complex **3**, Figure S5: Selection of the downfield region of HMQC spectrum of complex **3**, Figure S6: Kinetic profiles for *cis*-cyclooctene oxidation in the leaching experiment using material MCM-1. Figure S7: Self-assembly in the solid state of complexes **1** (view a) and **2** (view b) by C-H*···*O bonding contacts between carbonyl groups and C-H protons from allyl (**1**) or IMP (**2**) ligands. Figure S8: DFT calculated structures of the axial and equatorial isomers of complexes **1**, **2** and **3**, with selected distances (Å), Table S1: Characteristic ^1^H and ^13^C-NMR resonances (δ/ppm) for IMP, Me-IMP, Ph-IMP, and complexes **1**, **2** and **3**, Table S2: Crystal data of [Mo(η^3^-C_3_H_5_)Br(CO)_2_(IMP)] (**1**) and [Mo(η^3^-C_3_H_5_)Br(CO)_2_(Me-IMP)] (**2**).
