# Supplementary material for: New Molybdenum(II) Complexes with α-Diimine Ligands: Synthesis, Structure, and Catalytic Activity in Olefin Epoxidation
Source: Molecules. 2019 Feb 6;24(3):578. doi: 10.3390/molecules24030578 (PMC6384641; doi:10.3390/molecules24030578)
Supplement: Supplementary file 1 [file molecules-24-00578-s001.pdf]

Supporting Information
for

New molybdenum(II) complexes with α-diimine ligands: synthesis, structure, and catalytic activity in olefin epoxidation

Maria Vasconcellos-Dias [1], João Marreiros [1], Rita Sales [1], Vitor Félix [2], Paula Brandão [2], Carla D. Nunes [1,3], and Maria José Calhorda[1,4,*]

[1] Centro de Química e Bioquímica, DQB, Faculdade de Ciências, Universidade de Lisboa, Campo Grande, 1749-016 Lisboa, Portugal; mariavasconcellosdias@gmail.com; jtvrmarreiros@gmail.com; rituxa15@gmail.com

[2] Department of Chemistry, CICECO - Aveiro Institute of Materials, University of Aveiro, 3810-193 Aveiro, Portugal; vitor.felix@ua.pt; pbrandao@ua.pt

[3] Centro de Química Estrutural, Faculdade de Ciências, Universidade de Lisboa, 1049-001 Lisboa, Portugal; cmnunes@fc.ul.pt

[4] BioISI -Biosystems & Integrative Sciences Institute, Departamento de Química e Bioquímica, Faculdade de Ciências, Universidade de Lisboa, Campo Grande, 1749-016 Lisboa, Portugal; mjc@fc.ul.pt

* Correspondence: mjc@fc.ul.pt; Tel.: +351-217500196 (M.J.C.)

	Title	Page
**Figure S1**	NMR spectra of **1**: ^{1}H (bottom) and ^{13}C (top).	2
**Figure S2**	^{1}H NMR spectra of ligand Me-IMP (center), complex **2** (top), and ^{13}C NMR spectrum of complex **2**.	3
**Figure S3**	Selection of the upfield region of the bidimensional COSY spectrum of complex **3**.	4
**Figure S4**	Selection of the upfield region of the HMQC spectrum of complex **3**.	5
**Figure S5**	Selection of the downfield region of HMQC spectrum of complex **3**.	6
**Table S1**	Characteristic ^{1}H and ^{13}C NMR resonances (δ/ppm) for IMP, Me-IMP, Ph-IMP, and complexes **1**, **2** and **3**.	7
**Table S2**	Crystal data of [Mo(η^3-C_3H_5)Br$(CO)_2$(IMP)] (**1**) and [Mo(η^3-C_3H_5)Br$(CO)_2$(Me-IMP)] (**2**).	8
**Figure S6**	Self-assembly in the solid state of complexes **1** (view a) and **2** (view b) by C-H···O bonding contacts between carbonyl groups and C-H protons from allyl (**1**) or IMP (**2**) ligands..	9
**Figure S7**	DFT calculated structures of the axial and equatorial isomers of complexes **1**, **2** and **3**, with selected distances (Å).	10

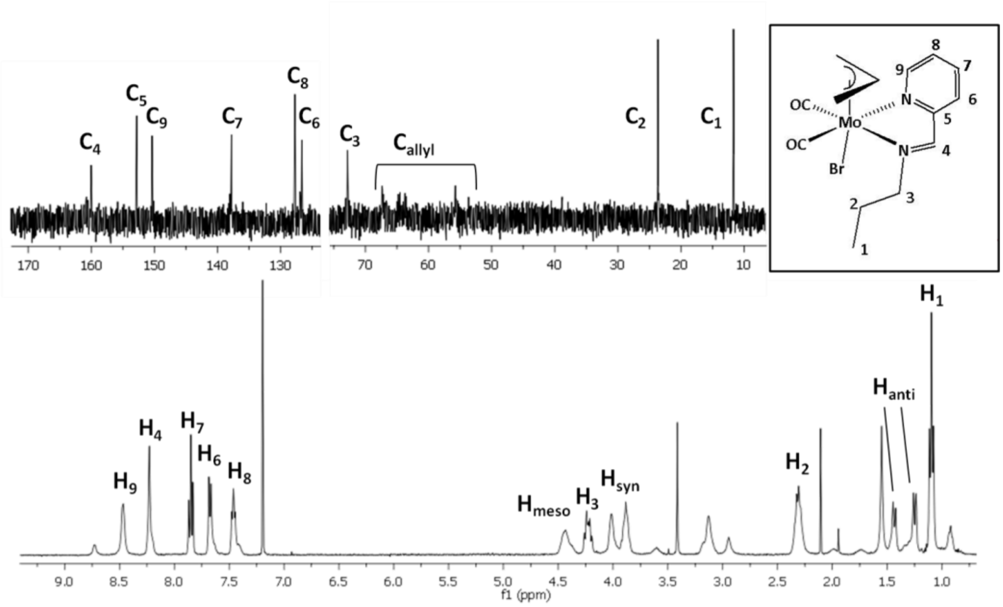

Figure S1. NMR spectra of **1**: ^{1}H (bottom) and ^{13}C (top).

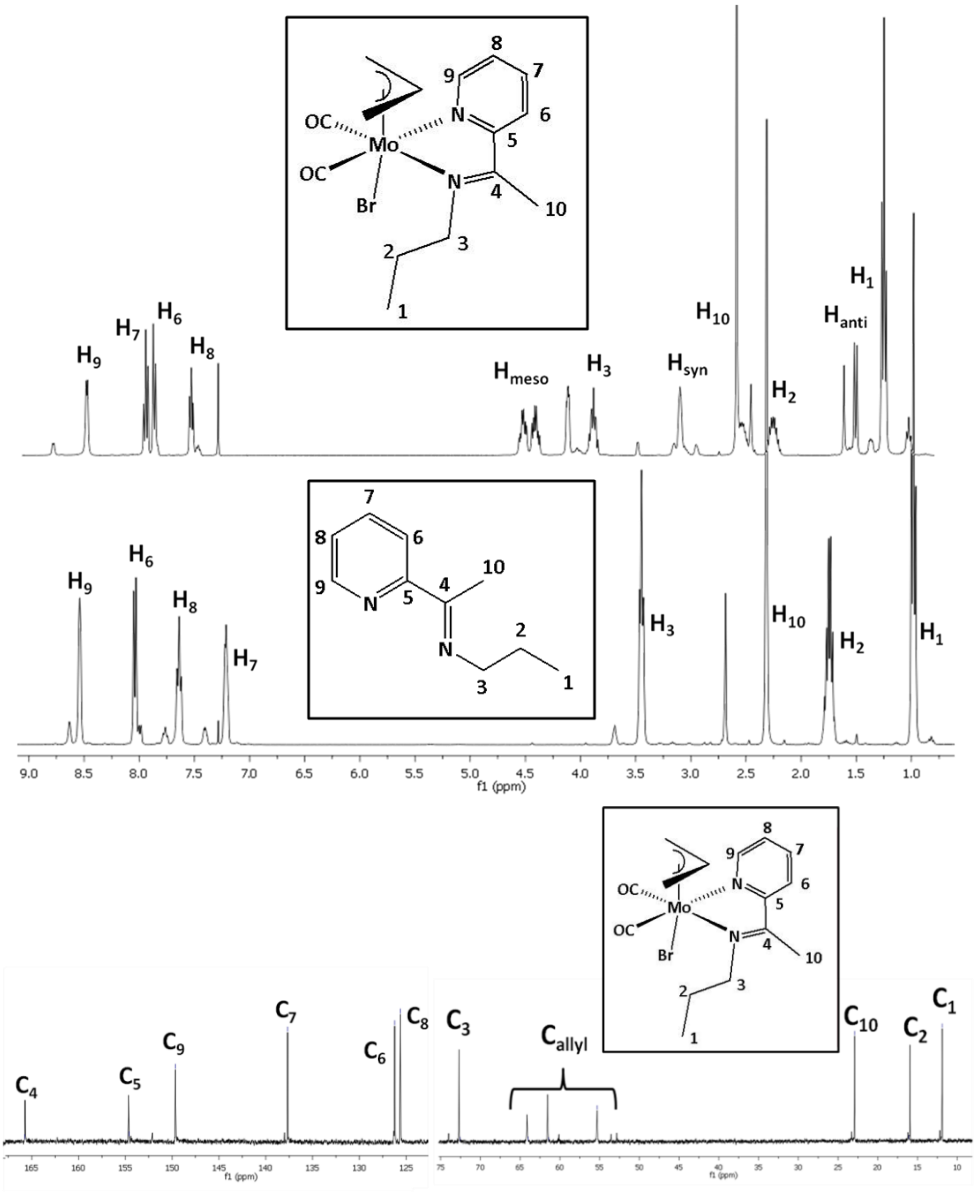

Figure S2. ^{1}H NMR spectra of ligand Me-IMP (center), complex **2** (top), and ^{13}C NMR spectrum of complex **2**.

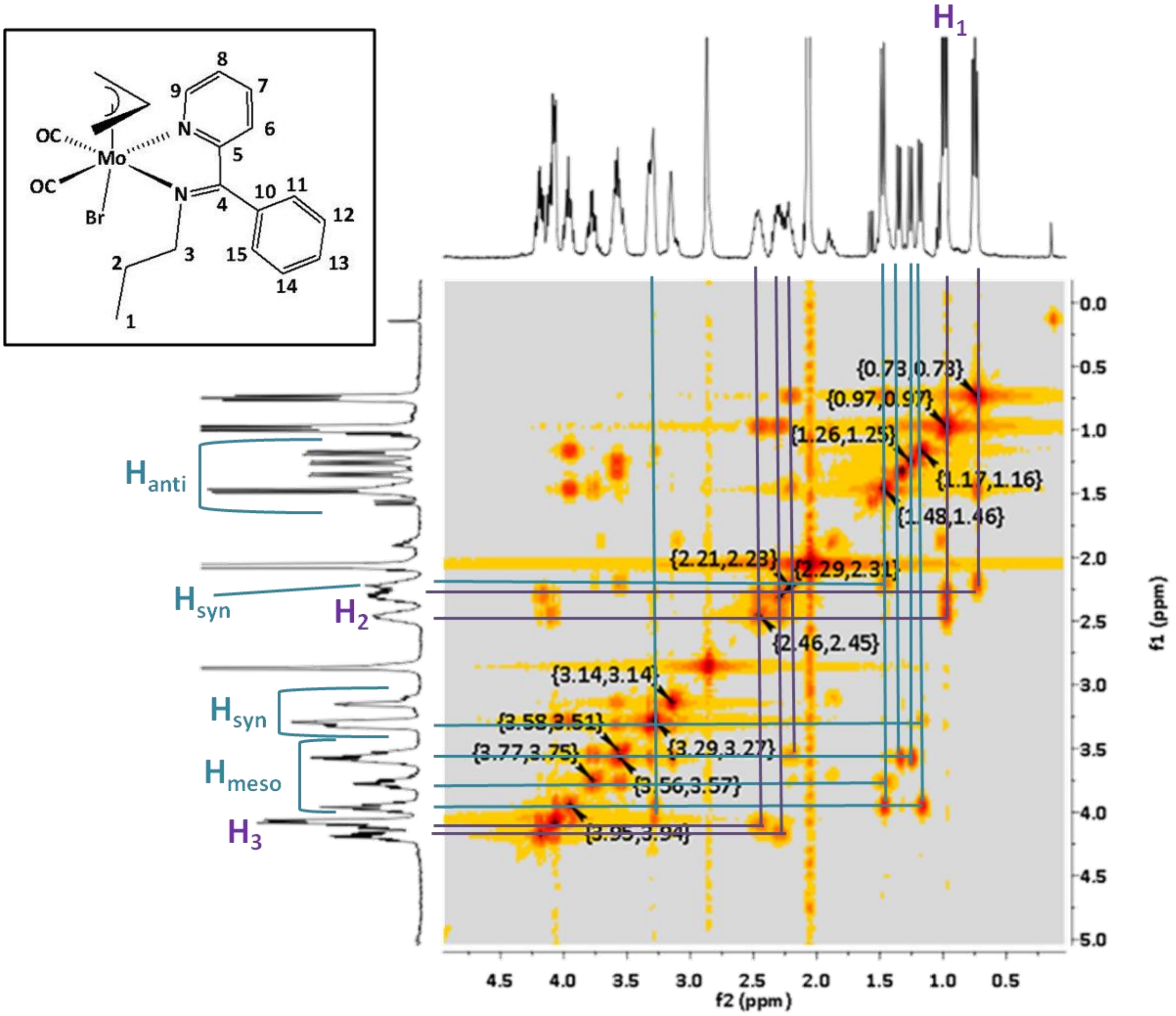

Figure S3. Selection of the upfield region of the bidimensional COSY spectrum of complex **3**.

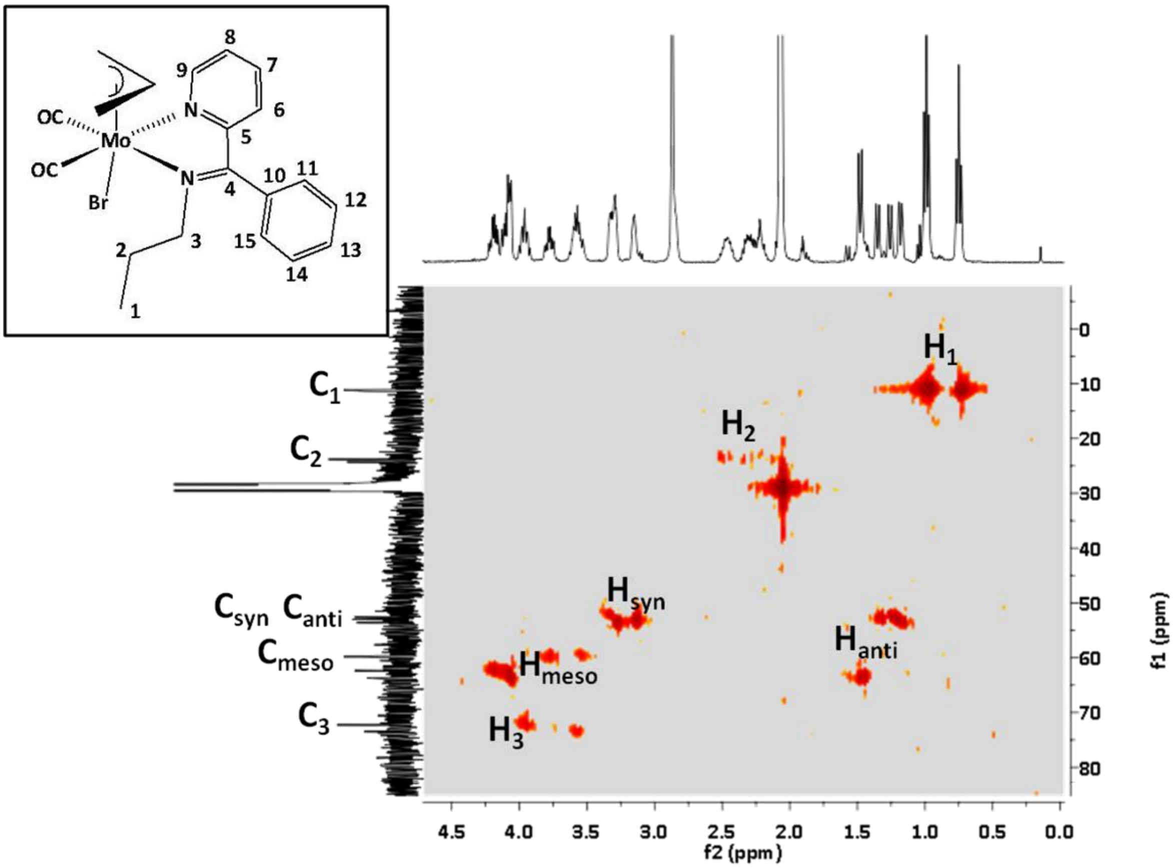

Figure S4. Selection of the upfield regionof the HMQC spectrum of complex **3**.

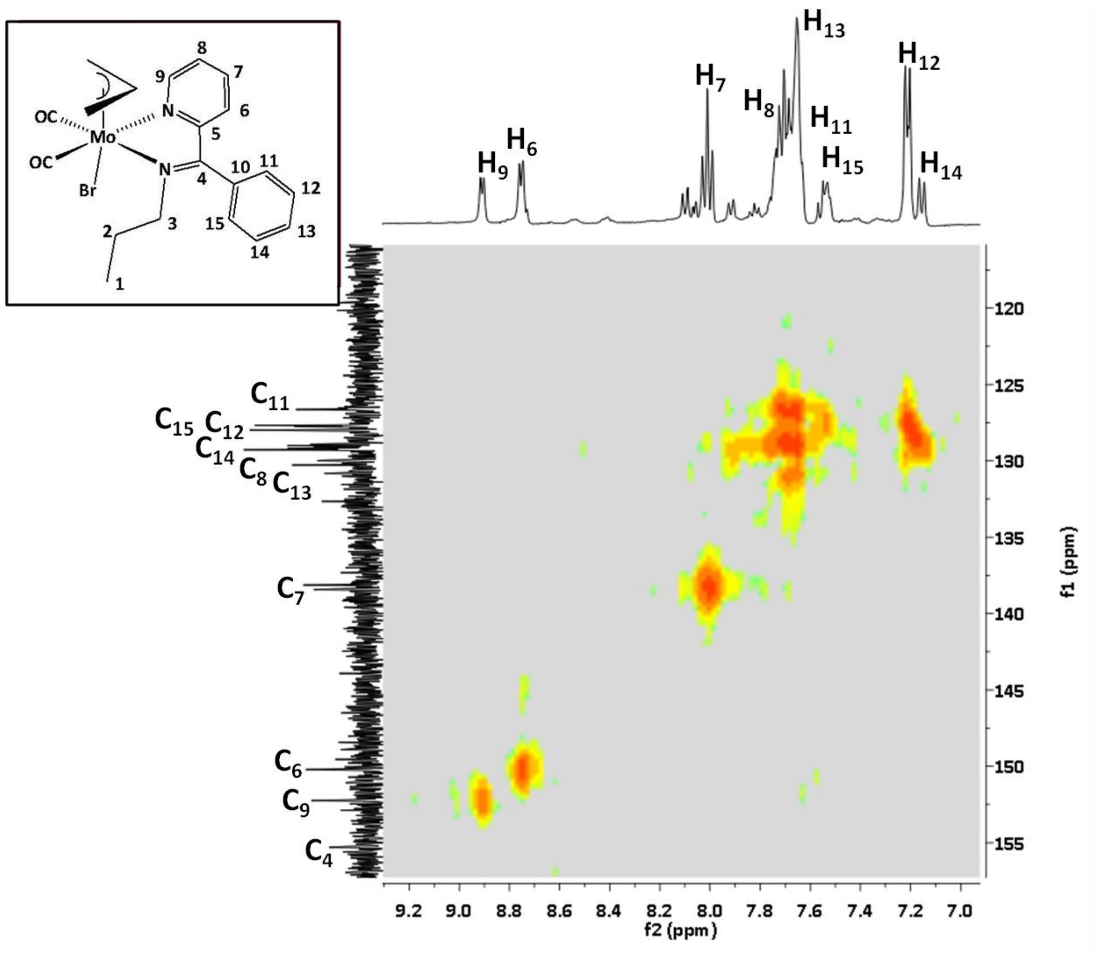

Figure S5. Selection of the downfield region of HMQC spectrum of complex **3**.

Table S1. Characteristic ^1H and ^{13}C NMR resonances (δ/ppm) for IMP, Me-IMP, Ph-IMP, and complexes **1**, **2** and **3**.

Compound	^{1}H NMR	^{13}C NMR
IMP	**H1** 0.98 **H2** 1.87 **H3** 3.79 **H4** 7.66	**C1** 11.6 **C2** 23.4 **C3** 61.6 **C4** 162.0
	**H6** 7.87 **H7** 7.61 **H8** 7.98 **H9** 8.70	**C5** 149.7 **C6** 125.3 **C7** 139.8 **C8** 127.6 **C9** 148.0
C1	**H1** 1.09 **H2** 2.31 **H3** 4.24 **H4** 8.24	**C1** 11.6 **C2** 23.5 **C3** 72.7 **C4** 160.2
	**H6** 7.69 **H7** 7.86 **H8** 7.48 **H9** 8.48	**C5** 152.7 **C6** 126.3 **C7** 137.7 **C8** 127.6
	$\mathbf{H_{anti}}$ 1.25 1.44 $\mathbf{H_{syn}}$ 2.95-4.00 $\mathbf{H_{meso}}$ 4.46	**C9** 150.2 $\mathbf{C_{allyl}}$ 55.1 $\mathbf{C_{allyl}}$ 68.4
Me-IMP	**H1** 0.96 **H2** 1.87 **H3** 3.56 **H6** 8.03	**C1** 11.9 **C2** 15.63 **C3** 54.2 **C4** 167.0
	**H7** 7.21 **H8** 7.64 **H9** 8.55 **H10** 2.40	**C6** 124.9 **C7** 142.1 **C8** 128.8 **C9** 146.4 **C10** 23.7
C2	**H1** 1.24 **H2** 2.25 **H3** 3.88 **H6** 7.87	**C1** 11.9 **C2** 16.2 **C3** 72.8 **C4** 165.7
	**H7** 7.94 **H8** 7.53 **H9** 8.47 **H10** 2.58	**C5** 154.6 **C6** 126.2 **C7** 125.6 **C8** 137.6 **C9** 150.1
	$\mathbf{H_{anti}}$ 1.49 1.61 $\mathbf{H_{syn}}$ 4.11 4.26 $\mathbf{H_{meso}}$ 4.41 4.80	**C10** 22.9 $\mathbf{C_{allyl\ term}}$ 55.2 61.5 $\mathbf{C_{allyl\ center}}$ 64.0
Ph-IMP	**H1** 0.69 **H2** 1.53 **H3** 3.13-3.22 **H6** 7.94	**C1** 10.7 **C2** 20.4 **C3** 41.0 **C4** 155.1 **C6** 124.7
	**H7** 8.02 **H8** 7.80 **H9** 8.63 **H11** **H15** 7.51	**C7** 137.0 **C8** 128.0 **C9** 148.3 **C11** **C15** 131.1
	**H12** 7.28 **H13** 7.41 **H14** 7.15	**C12** 126.1 **C13** 132.6 **C14** 135.9
C3	**H1** 0.73 0.97 **H2** 2.29 2.45 **H3** 4.08 **H6** 8.74	**C1** 11.0 **C2** 23.2 **C3** 72.0 **C4** 155.3 **C6** 150.3
	**H7** 8.01 **H8** 7.65 **H9** 8.91 **H11** **H15** 7.52	**C7** 138.2 **C8** 129.0 **C9** 152.2 **C12** 127.7 **C13** 130.9
	**H12** 7.20 **H13** 7.72 **H14** 7.20	**C14** 128.9 **C11** 126.6 **C15** 128.9 $\mathbf{C_{anti}}$ 52.7 53.2 63.5
	$\mathbf{H_{anti}}$ 1.16 1.25 1.34 1.48 $\mathbf{H_{syn}}$ 3.14 3.28 $\mathbf{H_{meso}}$ 3.58 3.76 3.95	$\mathbf{C_{syn}}$ 52.9 53.0 59.6 $\mathbf{C_{meso}}$ 62.6 73.3

Table S2. Crystal data of [Mo(η^3-C_3H_5)Br$(CO)_2$(IMP)] (**1**) and [Mo(η^3-C_3H_5)Br$(CO)_2$(Me-IMP)] (**2**).

Complex	**1**	**2**
Empirical formula	$C_{28}H_{34}Br_2Mo_2N_4O_4$	$C_{15}H_{19}BrMoN_2O_2$
Formula weight	842.29	435.17
Crystal system	Triclinic	Monoclinic
Space group	$P\bar{1}$	$P2_1/n$
α (Å)	10.6531(4)	12.0236(4)
b (Å)	10.7560(5)	9.0449(3)
c (Å)	14.0254(7)	14.9858(4)
α (°)	87.607(2)	(90)
β (°)	75.561(2)	104.382(3)
γ (°)	81.487(2)	(90)
Volume (Å^3)	1539.17(12)	1626.97(9)
Z	4	4
ρ_{calc} (g/cm^3)	1.817	1.777
μ (mm^{-1})	3.450	3.267
F(000)	832	864
Index ranges	$-16 \leq h \leq 15$, $-16 \leq k \leq 16$, $-21 \leq l \leq 21$	$-16 \leq h \leq 16$, $-12 \leq k \leq 12$, $-20 \leq l \leq 18$
Reflections collected	42861 ($4.36 \leq 2\theta \leq 66.58$ °)	29346 ($5.26 \leq 2\theta \leq 58.52$ °)
Unique reflections, [R_{int}]	11254 [0.0266]	4392 [0.0236]
Final R indexes		
R_1, wR_2 [I>2σI]	0.0222, 0.0478 [9481]	0.0165, 0.0444 [4086]
R_1, wR_2 (all data)	0.0323, 0.0505	0.0190, 0.0454
Data/restraints/ parameters	11254/0/395	4392/0/208
Goodness-of-fit on F^2	1.025	1.029
Largest diff. peak/hole (eÅ^{-3})	0.99/-0.56	0.39/-0.35

a)

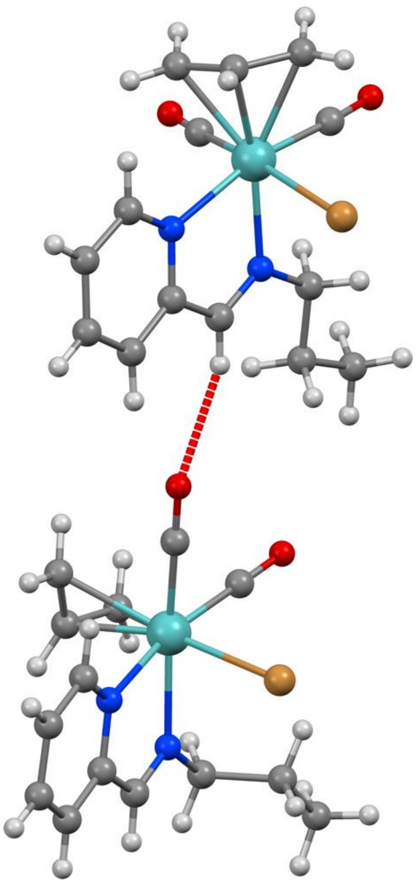

b)

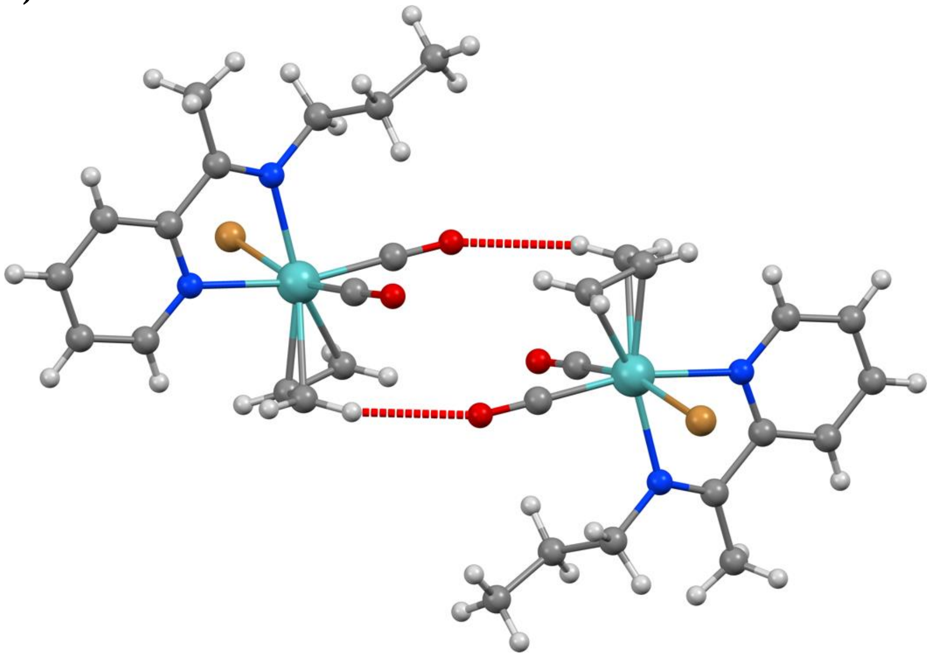

Figure S6. Self-assembly in the solid state of complexes **1** (view a) and **2** (view b) by C-H ··O bonding contacts between carbonyl groups and C-H protons from allyl (**1**) or IMP (**2**) ligands.

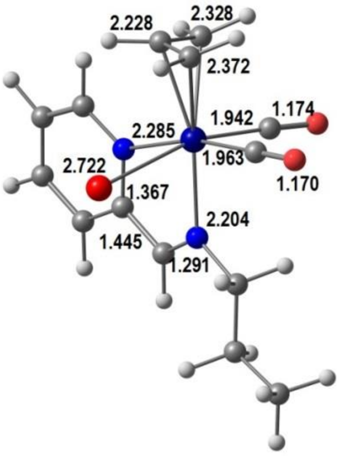

1 (axial)

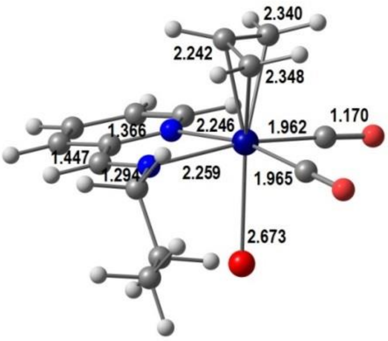

1 (equatorial)

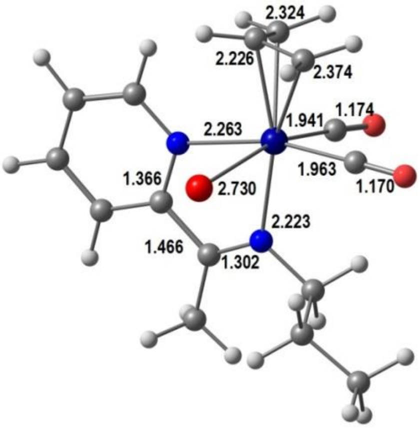

2 (axial)

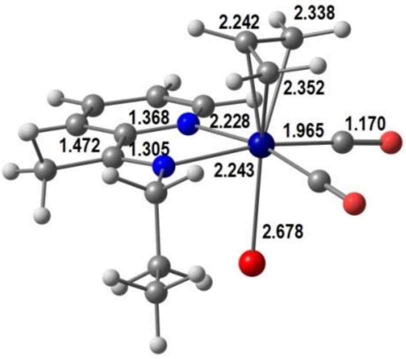

2 (equatorial)

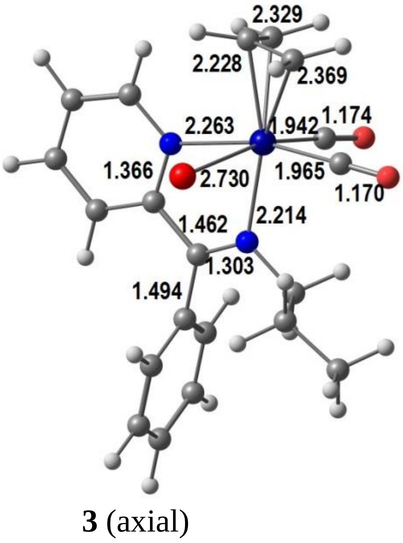

3 (axial)

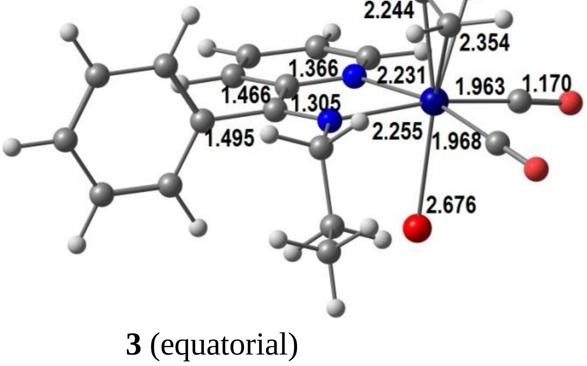

3 (equatorial)

Figure S7. DFT calculated structures of the axial and equatorial isomers of complexes **1**, **2** and **3**, with selected distances (Å).
